# Supplementary figures and images for: Active removal of inhibitory components drives the flagellar type 3 secretion-specificity switch
Source: mBio. 2026 Jun 9;17(7):e01037-26. doi: 10.1128/mbio.01037-26 (PMC13344023; doi:10.1128/mbio.01037-26)

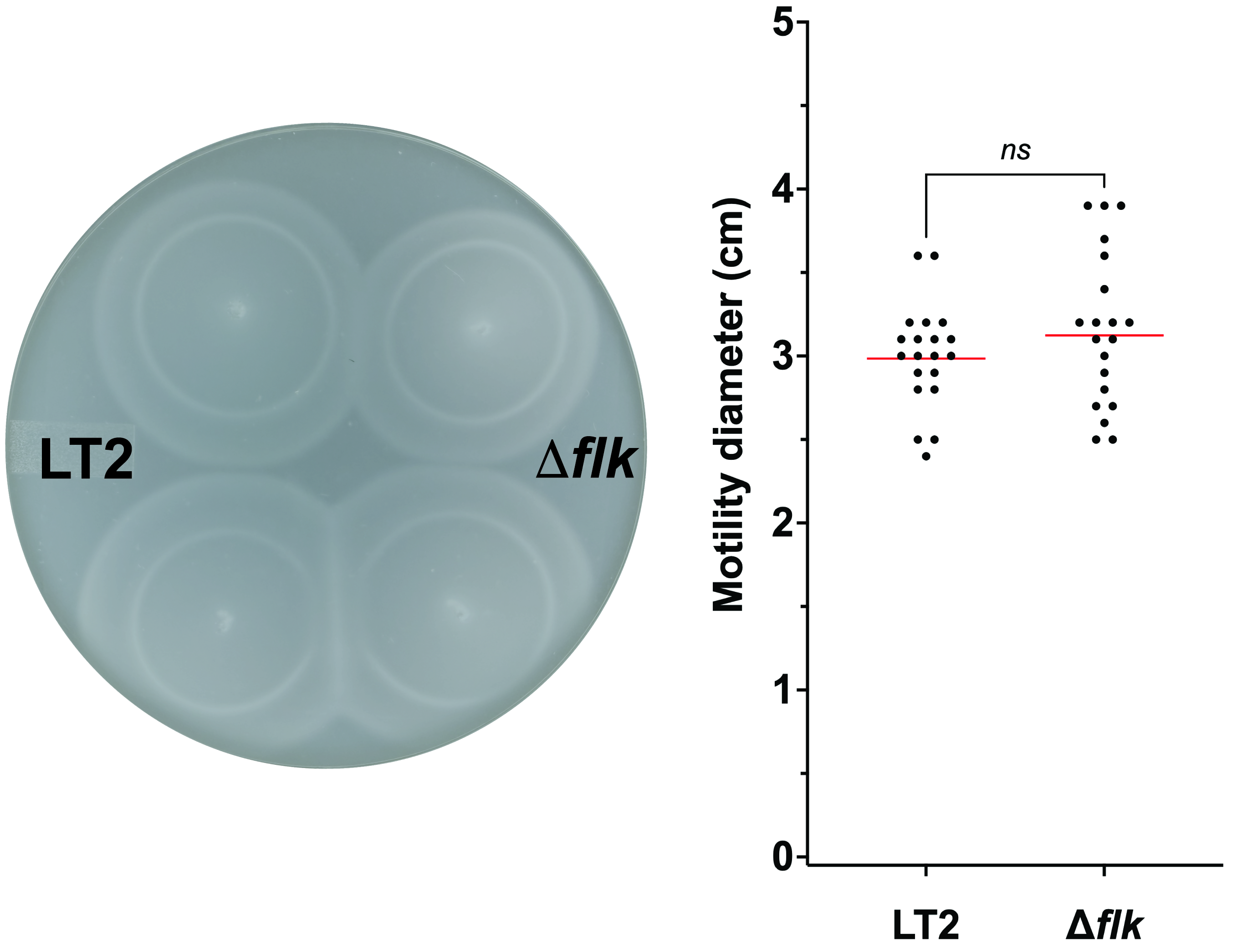

Supplement: Fig. S1 — The deletion of flk has no apparent effect on Salmonella motility. [file mbio.01037-26-s0001.tif]

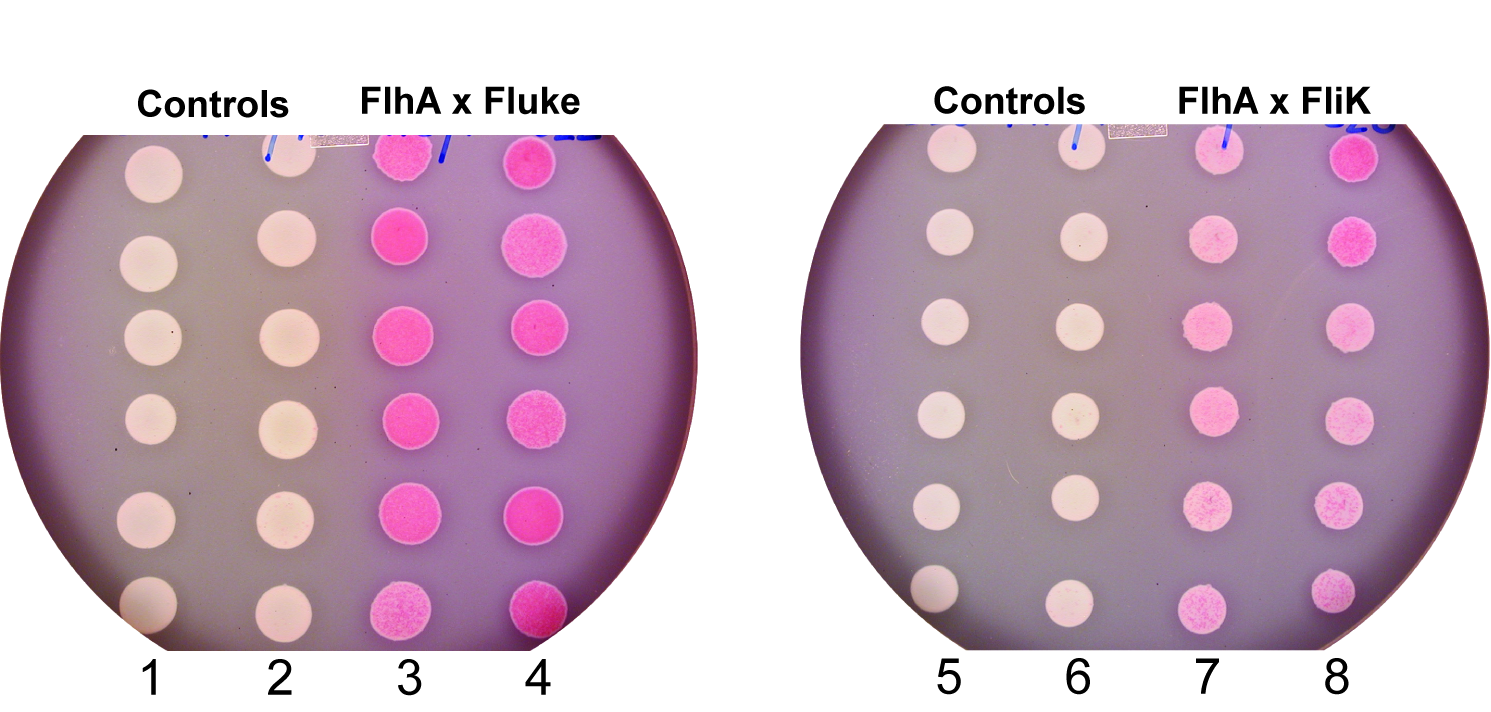

Supplement: Fig. S2 — Bacterial two-hybrid screen for interaction between Fluke or FliK and FlhAC. [file mbio.01037-26-s0002.tif]

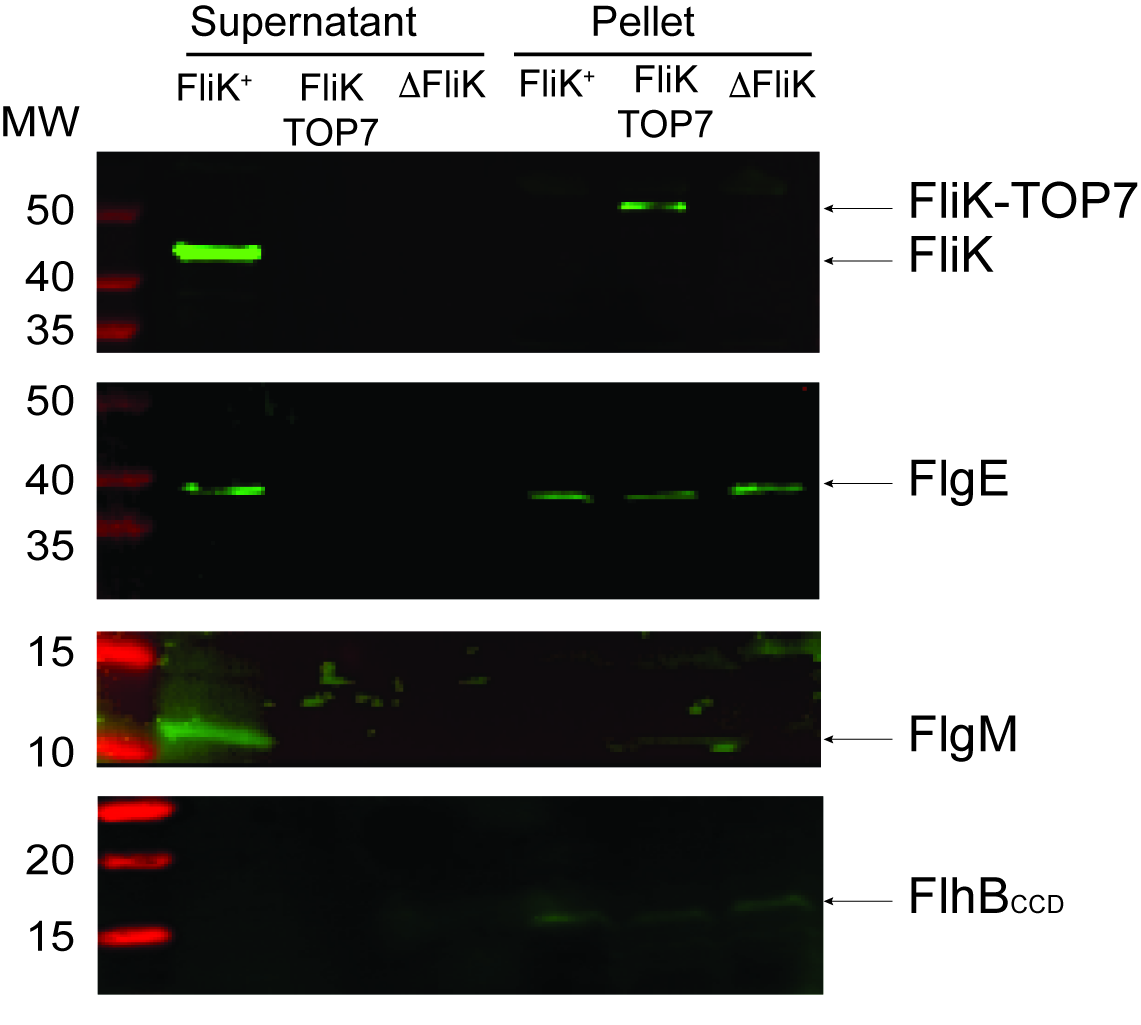

Supplement: Fig. S3 — The cleavable C-terminal domain of FlhB is not detected in the cell supernatant. [file mbio.01037-26-s0003.tif]

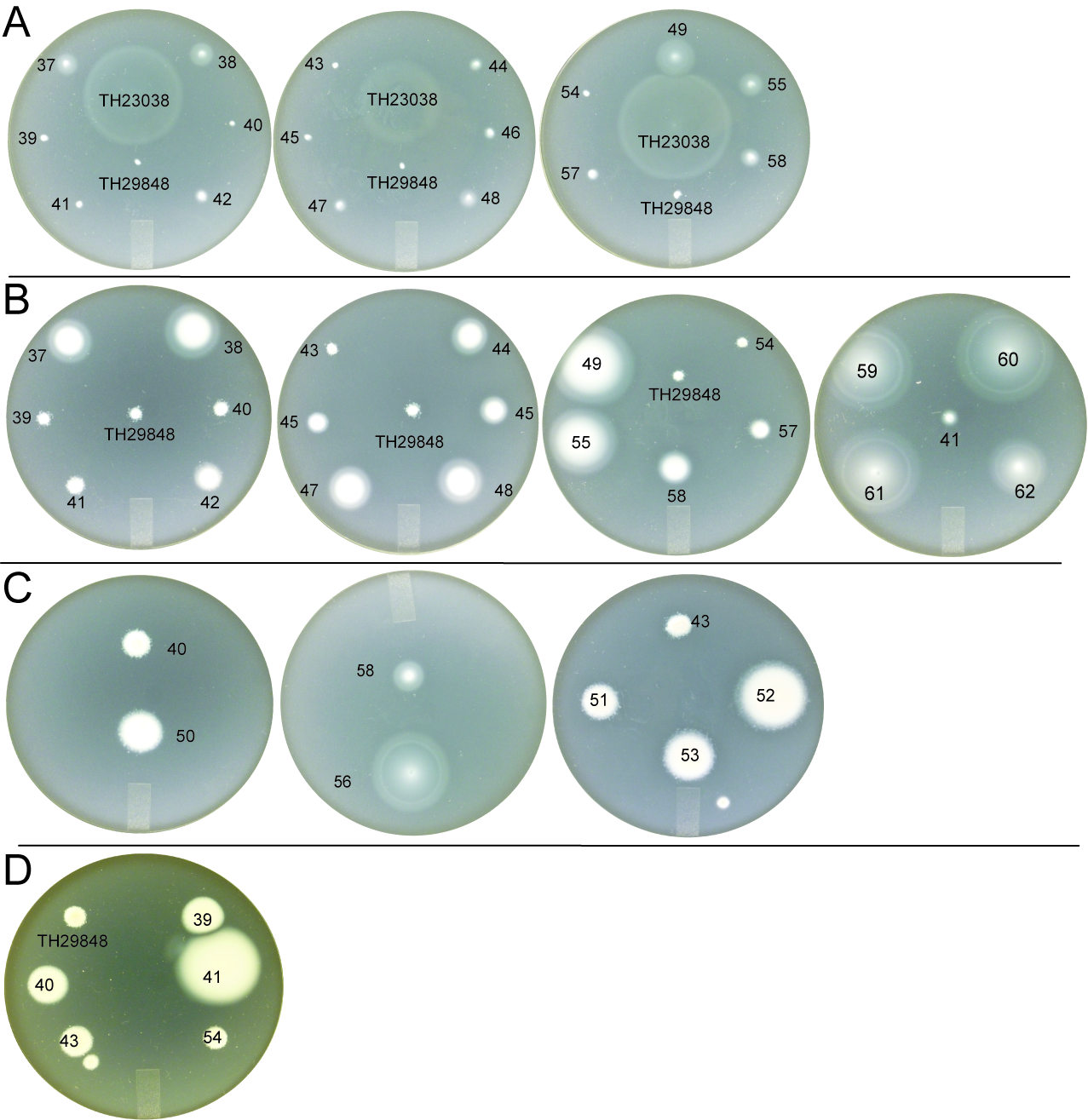

Supplement: Fig. S4 — Motile revertants of ΔfliK Δflk double mutant strain in the flhD*C* background. [file mbio.01037-26-s0004.tif]

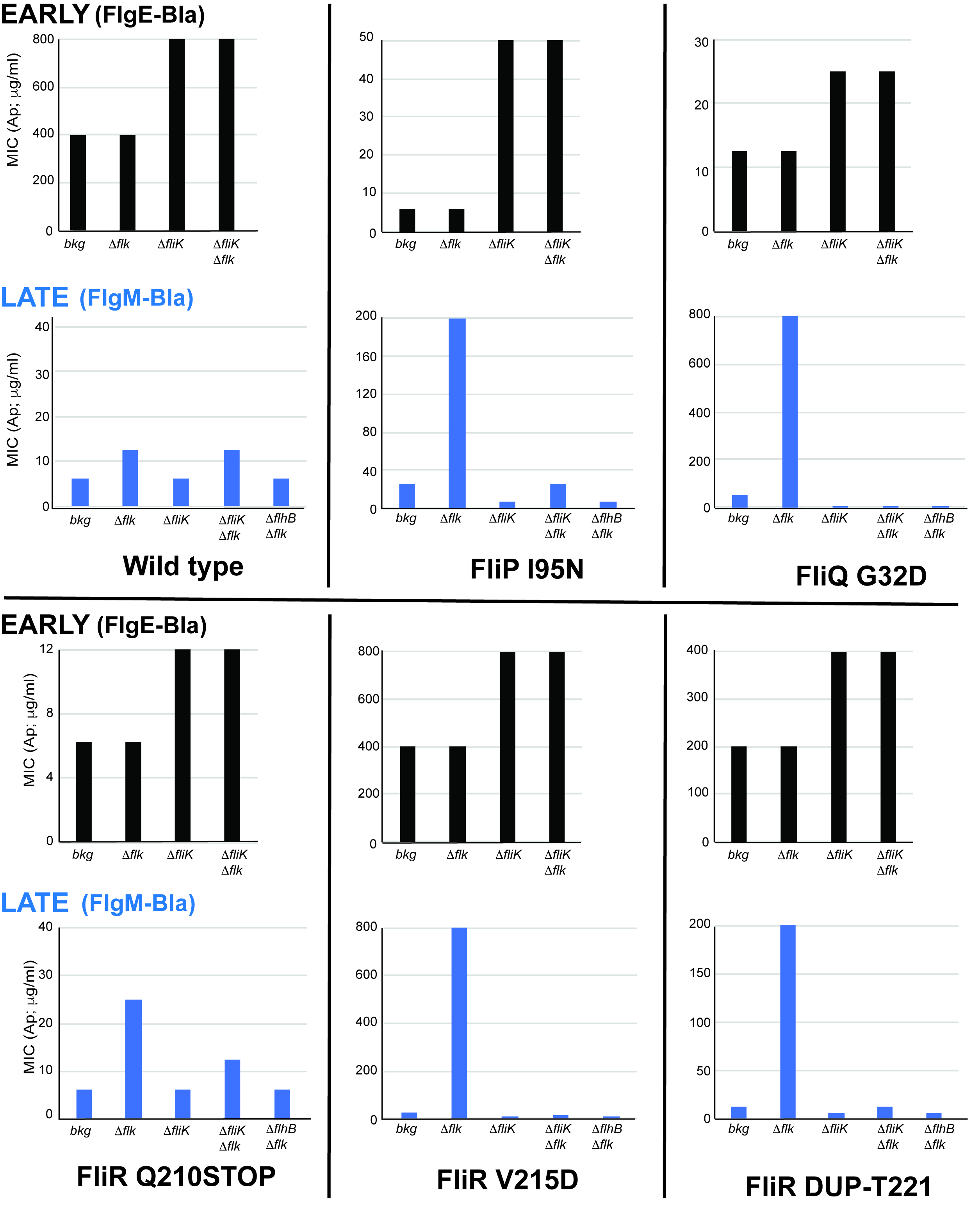

Supplement: Fig. S5 — Early and late substrate secretion in the FlhB-bypass mutants in the presence and absence of Fluke and/or FliK. [file mbio.01037-26-s0005.tif]
