# Supplementary material for: Active removal of inhibitory components drives the flagellar type 3 secretion-specificity switch
Source: mBio. 2026 Jun 9;17(7):e01037-26. doi: 10.1128/mbio.01037-26 (PMC13344023; doi:10.1128/mbio.01037-26)
Supplement: Supplemental Material — Supplemental tables and legends for supplemental figures. [file mbio.01037-26-s0006.docx]

**Supplemental Tables**

### **Table S1. Plasmids and Bacterial strains used in this study***

**Plasmids:**

| Plasmid Name | Usage and relevant characteristic | Source* |
| --- | --- | --- |
| pKD3 | For inserting FRT-Cm-FRT chromosomal cassette, Ap^R^ plasmid | (13) |
| p-Sim5 | Temperature inducible λ-Red expressing plasmid, Cm^R^ plasmid, pSC101 origin of replication (replicates at 30°C) | (14) |
| p-Sim-6 | Temperature inducible λ-Red expressing plasmid, Ap^R^ plasmid, pSC101 origin of replication (replicates at 30°C) | (14) |
| p-EM5 | Km^R^ P*_nahG_*-*fliQ*^+^ | M. Erhardt |

***E.coli* strains:**

| ***E.coli* Strains** | Genotype | Source* |
| --- | --- | --- |
| MG1655MGRFC | Δ*leuB* *motA*(E98K) Int5::Zeo^R^-PRNA-mcherry *galK*::P*_fliF_*(*E. coli*)-CFP-Ap^R^ *attB*::P*_fliC_*(*E. coli*)-YFP-Km^R^ | (33) |
| TH408 | *nad*::Tn*10d*Tc | Lab collection |
| BTH101 | F^-^ *cya-99 araD139 galE15 galK16 rpsL1*(Str^R^) *hsdR2 mcrA1 mcrB1* | BACTH, Euromedex |

***Salmonella* Typhimurium strains:**

| *Salmonella* strains | Genotype | Source* |
| --- | --- | --- |
| TH437 | LT2 *Salmonella* wild type strain | J.Roth |
| TH9949 | *flg*E*6569*::*bla* Δ*flgBC6557* | Lab collection |
| TH13359 | *fliK7582*::*bla* | Lab collection |
| TH13807 | *fliK7582*::*bla* Δ*flgA7656* (Δamino acids 6-214 (of 219)) | Lab collection |
| TH13808 | *fliK7582*::*bla* Δ*flgB7657* (Δamino acids 6-133(of 138)) | Lab collection |
| TH13809 | *fliK7582*::*bla* Δ*flgC7658* (Δamino acids 6-129(of 134)) | Lab collection |
| TH13810 | *fliK7582*::*bla* Δ*flgD6540* (Δamino acids 2-220(of 232)) | Lab collection |
| TH13811 | *fliK7582*::*bla* Δ*flgE7659* (Δamino acids 6-398(of 403)) | Lab collection |
| TH13812 | *fliK7582*::*bla* Δ*flgF7660* (Δamino acids 6-246(of 251)) | Lab collection |
| TH13813 | *fliK7582*::*bla* Δ*flgG7661* (Δamino acids 6-255(of 260)) | Lab collection |
| TH13814 | *fliK7582*::*bla* Δ*flgH7662* (Δamino acids 6-227(of 232)) | Lab collection |
| TH13815 | *fliK7582*::*bla* Δ*flgI7663* (Δamino acids 6-360(of 365)) | Lab collection |
| TH13816 | *fliK7582*::*bla* Δ*flgJ7664* (Δamino acids 6-311(of 316)) | Lab collection |
| TH13817 | *fliK7582::bla* Δ*flgK7665* (Δamino acids 6-548(of 553)) | Lab collection |
| TH13818 | *fliK7582*::*bla* Δ*flgL7666* (Δamino acids 6-312(of 317)) | Lab collection |
| TH14607 | Δ*flk-7755* | Lab collection |
| TH23959 | Δ*flhB8612*_ΔCCD_ (*flhB*_ΔCCD_ = deletion of amino acids P270-G383) |  |
| TH24321 | Δ*flgBC6557* *flgE6569*::*bla* Δ*flk-7755* | Lab collection |
|  |  |  |

| *Salmonella* strains | Genotype | Source* |
| --- | --- | --- |
| TH24722 | *flgM6427::bla* Δ*flgB-L8735* Δ*flhB8612*_ΔCCD_ *flhD8070* *flhC8092* *fliA5225*(H14D) Δ*fliB-T7771* *fljB^enx^* *vh2* |  |
| TH24787 | *flgM6427*::*bla* Δ*flgB-L8735* Δ*flhB8612*_ΔCCD_ *flhD8070 flhC8092 fliA5225*(H14D) Δ*fliB-T7771* Δ*flk-7755 fljB^enx^ vh2* |  |
| TH24789 | Δ*araBAD961*::*flk^+^ flgM6427::bla* Δ*flgB-L8735* Δ*flhB8612*_ΔCCD_ *flhD8070 flhC8092 fliA5225*(H14D) Δ*fliB-T7771 fljB^enx^ vh2* |  |
| TH24800 | *flgM6427*::*bla* Δ*flgB-L8735* Δ*flhB8612*_ΔCCD_ *flhD8070 flhC8092 fliA5225*(H14D) Δ*fliB-T7771* Δ*fliK6140* Δ*flk-7755 fljB^enx^ vh2* |  |
| TH24819 | *flgM6427*::*bla* Δ*flgB-L8735* Δ*flhB8612*_ΔCCD_ *flhD8070 flhC8092 fliA5225*(H14D) Δ*fliB-T7771* Δ*fliK6140* *fljB^enx^ vh2* |  |
| TH25226 | *flgM6427*::*bla* Δ*flgB-L8735* *flhD8070 flhC8092* *fliA5225*(H14D) Δ*fliB-T7771* Δ*flk-7755* *fljB^enx^* *vh2* |  |
| TH25935 | *flgM6427*::*bla* ΔflgB-L8735 *flhB8612*_ΔCCD_ *fljB^enx^* *vh2* |  |
| TH25936 | Δ*flgBC6557* *flgE6569*::*bla* *flhB861*_ΔCCD_ *2* *fljB^enx^* *vh2* |  |
| TH25938 | *flgM6427*::*bla* Δ*flgB-L8735* *flhB8612*_ΔCCD_ Δ*flk-7755* *fljB^enx^* *vh2* |  |
| TH25939 | Δ*flgBC6557* *flgE6569*::*bla* *flhB8612*_ΔCCD_ Δ*flk-7755* *fljB^enx^* vh2 |  |
| TH26028 | *flgM6427*::*bla* Δ*flgB-L8735* *flhB8612*_ΔCCD_ *fliK6620* Δ*flk-7755* *fljB^enx^* *vh2* |  |
| TH26992 | *flgM6427*::*bla* Δ*flgB-L8735* *STM1911*::Tn*10d*Tc Δ*flhBAE7670*::FCF *flhD8070 flhC8092* *fliA5225*(H14D) Δ*fliB-T7771* Δ*flk-7755 fljB^enx^* *vh2* |  |
| TH27083 | pSIM5/LT2 |  |
| TH27122 | Δ*araBAD957*::*rflP^+^* Δ*rflM8403* P*_flh_*_DC_*5451*::TPOP *fljB^enx^* *vh2* |  |
| TH27152 | *flgM6427*::*bla* Δ*flgB-L8735* *flhD8070 flhC8092* *fliA5225*(H14D) Δ*fliB-T7771* Δ*fliQ8223*::*tetRA* (Δ codons 31-50) Δflk-7755 *fljB^enx^* *vh2* |  |
| TH27337 | *attB*::Cm^R^-P*_fliF_*-YFP *argW*::Zeo^R^-mcherry Δ*galK*::Ap^R^-P*_fliC_*-CFP P*_flhDC_5451*::TPOP *fljB^enx^* *vh2* |  |
| TH27343 | *attB*::Cm^R^-P*_fliF_*-YFP *argW*::Zeo^R^-mcherry Δ*galK*::Ap^R^-P*_fliC_*-CFP *flgM7929*::TPOP *fljB^enx^* *vh2* |  |
| TH27622 | P*_flhDC_5451*::TPOP Δ*rflM8403* *fljB^enx^* *vh2* |  |
| TH27623 | P*_flhDC_5451*::TPOP Δ*rflM8403* Δ*fliK6140* *fljB^enx^* *vh2* |  |
| TH27965 | Δ*araBAD2101*::*flhB*^+^ *flgM6427*::*bla* Δ*flgB-L8735* *flhD8070 flhC8092 fliA5225*(H14D) Δ*fliB-T7771* Δ*flk-7755 fljB^enx^ vh2* |  |
| TH28049 | *attB*::Cm^R^-P*_fliF_*-YFP Δ*galK*::Ap^R^-P*_fliC_*-CFP *fljB^enx^* *vh2* |  |
| TH28050 | *attB*::Cm^R^-P*_fliF_*-YFP Δ*galK*::Ap^R^-P*_fliC_*-CFP Δ*flgHI958 fljB^enx^ vh2* |  |
| TH28051 | *attB*::Cm^R^-P*_fliF_*-YFP Δ*galK*::Ap^R^-P*_fliC_*-CFP *flhB8612 fljB^enx^ vh2* |  |
| TH28052 | *attB*::Cm^R^-P*_fliF_*-YFP Δ*galK*::Ap^R^-P*_fliC_*-CFP Δ*flk-7755* *fljB^enx^* *vh2* |  |
| TH28053 | *attB*::Cm^R^-P*_fliF_*-YFP Δ*galK*::Ap^R^-P*_fliC_*-CFP Δ*flgHI958* Δ*flk*-*7755* *fljB^enx^* *vh2* |  |
| TH28054 | *attB*::Cm^R^-P*_fliF_*-YFP Δ*galK*::Ap^R^-P*_fliC_*-CFP *flhB8612*_ΔCCD_ Δ*flk-7755 fljB^enx^ vh2* |  |
| TH28055 | *attB*::Cm^R^-P*_fliF_*-YFP Δ*galK*::Ap^R^-P*_fliC_*-CFP *flhB8612*_ΔCCD_ Δ*flgHI958* *fljB^enx^* *vh2* |  |
| TH28056 | *attB*::Cm^R^-P*_fliF_*-YFP Δ*galK*::Ap^R^-P*_fliC_*-CFP *flhB8612*_ΔCCD_ Δ*flgHI958* Δ*flk-7755 fljB^enx^* *vh2* |  |
| TH28157 | *flgM642*7::*bla* Δ*flgB-L8735* *fljB^enx^* *vh2* |  |
| TH28158 | *flgM6427*::*bla* Δ*flgB-L8735* Δ*flk-7755* *fljB^enx^* *vh2* |  |
|  |  |  |
| *Salmonella* strains | Genotype | Source* |
| TH29063 | Δ*araBAD2101*::*flhB^+^* *flgM6427*::*bla* Δ*flgB-L8735* *flhB8612*_ΔCCD_ Δ*flk-7755* *fljB^enx^* *vh2* |  |
| TH29064 | Δ*araBAD2101*::*flhB^+^* Δ*flgBC6557* *flgE6569*::*bla* *flhB8612*_ΔCCD_ Δ*flk-7755* *fljB^enx^* *vh2* |  |
| TH29116 | Δ*flgBC6557* *flgE6569*::*bla* Δ*flhB8612*_ΔCCD_ Δ*fliK9249* Δ*flk-7755* |  |
| TH29314 | *attB*::Cm^R^-P*_fliF_*-YFP Δ*galK*::Ap^R^-P*_fliC_*-CFP Δ*flk-7755* *fljB^enx^* *vh2* |  |
| TH29315 | *attB*::Cm^R^-P*_fliF_*-YFP Δ*galK*::Ap^R^-P*_fliC_*-CFP *flhB7152*(N269A) Δ*flgHI958* *fljB^enx^* *vh2* |  |
| TH29316 | *attB*::Cm^R^-P*_fliF_*-YFP Δ*galK*::Ap^R^-P*_fliC_*-CFP *flhB7152*(N269A) Δ*flgHI958* Δ*flk-7755* *fljB^enx^* *vh2* |  |
| TH29317 | *attB*::Cm^R^-P*_fliF_*-YFP Δ*galK*::Ap^R^-P*_fliC_*-CFP *flhB8612*_ΔCCD_ *flhD8070* *flhC8092* Δ*flk-7755* *fljB^enx^* *vh2* |  |
| TH29318 | *attB*::Cm^R^-P*_fliF_*-YFP Δ*galK*::Ap^R^-P*_fliC_*-CFP *flhB8612*_ΔCCD_ *flhD8070* *flhC8092* Δ*flgHI958 fljB^enx^* *vh2* |  |
| TH29319 | *attB*::Cm^R^-P*_fliF_*-YFP Δ*galK*::Ap^R^-P*_fliC_*-CFP *flhB8612*_ΔCCD_ *flhD8070* *flhC8092* Δ*flgHI958* Δ*flk-7755* *fljB^enx^* *vh2* |  |
| TH29357 | *attB*::Cm^R^-P*_fliF_*-YFP Δ*galK*::Ap^R^-P*_fliC_*-CFP Δ*flhB8789 (*leaves first 5 and last 9 amino acids of FlhB) *flhD8070* *flhC8092* Δ*flk-7755* *fljB^enx^* *vh2* |  |
| TH29358 | *attB*::Cm^R^-P*_fliF_*-YFP Δ*galK*::Ap^R^-P*_fliC_*-CFP Δ*flgHI958* *flhB8789* *flhD8070 flhC8092* *fljB^enx^* *vh2* |  |
| TH29359 | *attB*::Cm^R^-P*_fliF_*-YFP Δ*galK*::Ap^R^-P*_fliC_*-CFP Δ*flgHI958* *flhB8789* *flhD8070* *flhC8092* Δ*flk-7755* *fljB^enx^* *vh2* |  |
| TH29848 | *flhD8070* *flhC8092* Δ*fliK9249* Δ*flk-7755* |  |
| TH30093 | Δ*ara*B*AD7606*::*fliK*^+^ Δ*fliK9249 attB*::Cm^R^-P*_fliF_*-YFP Δ*galK*::Ap^R^-P*_fliC_*-CFP *flhD8070* *flhC8092* Δ*prgH73*::*tetRA* *ompT*::Km *fljB^enx^* *vh2* |  |
| TH30110 | Δ*araBAD7609*::*fliK-*TOP7 Δ*fl*iK9249 *attB*::Cm^R^-P*_fliF_*-YFP Δ*galK*::Ap^R^-P*_fliC_*-CFP *flhD8070* *flhC8092* Δ*prgH73*::*tetRA* *ompT*::Km *fljB^enx^* *vh2* |  |
| TH30111 | Δ*araBAD937*::FKF Δ*fliK9249 attB*::Cm^R^-P*_fliF_*-YFP Δ*galK*::Ap^R^-P*_fliC_*-CFP *flhD8070* *flhC8092* Δ*prgH73*::*tetRA* *ompT*::Km *fljB^enx^* *vh2* |  |
| TH30250 | Δ*flgBC6557* *flgE6569*::*bla* Δ*flhB8612*_ΔCCD_ Δ*fliK9249* |  |
| TH30367 | *fliK9504*(Δaa311-320) *fliK7582*::*bla* |  |
| TH30368 | *fliK9505*(P296L) *fliK7582*::*bla* |  |
| TH30413 | Δ*galK*::P*_fliC_*-*luxCDBAE*-Cm^R^ P*_flhDC_5451*::Tn*10d*Tc[*del-25*] *fliK7582*::*bla* |  |
| TH30414 | Δ*galK*::P*_fliC_*-*luxCDBAE*-Cm^R^ P*_flhDC_5451*::Tn*10d*Tc[*del-25*] *fliK9504*(Δaa311-320) *fliK7582*::*bla* |  |
| TH30415 | Δ*galK*::P*_fliC_*-*luxCDBAE*-Cm^R^ P*_flhDC_5451*::Tn*10d*Tc[*del-25*] *fliK9505*(P296L) *fliK7582*::*bla* |  |

### *Unless indicated otherwise these strains were constructed during this work

***Salmonella enterica serovar Typhimurium* 14028** **strains:**

| Salmonella strains | Genotype | Source* |
| --- | --- | --- |
| TH27356 | 14028 *argW*::Zeo^R^-PmCherry *attB*::Km^R^-P*_fliF_*-YFP Δ*galK*::Ap^R^-P*_fliC_*-CFP Δ*prgH74* Δ*ssaN109* Δ*flgHI958* *motA8739*(E98K) Δ*fliC7716* *fljB^enx^* *vh2* |  |
| TH27359 | 14028 *argW*::Zeo^R^-PmCherry *attB*::Km^R^-P*_fliF_*-YFP Δ*galK*::Ap^R^-P*_fliC_*-CFP Δ*prgH74* Δ*ssaN109* Δ*flgHI958* Δ*flk*-7755 *motA8739*(E98K) Δ*fliC7716* *fljB^enx^* *vh2* |  |
| TH27476 | 14028 *argW*::Zeo^R^-PmCherry *attB*::Km^R^-P*_fliF_*-YFP Δ*galK*::Ap^R^-P*_fliC_*-CFP Δ*prgH74* Δ*ssaN109* Δ*flgHI958* Δ*flk*-7755 *flhB8612*_ΔCCD_ *motA8739* Δ*fliC7716* *fljB^enx^* *vh2* |  |
| TH27477 | 14028 *argW*::Zeo^R^-PmCherry *attB*::Km^R^-P*_fliF_*-YFP Δ*galK*::Ap^R^-P*_fliC_*-CFP Δ*prgH74* Δ*ssaN109* Δ*flgHI958 flhB8612* *motA8739* Δ*fliC7716* *fljB^enx^* *vh2* |  |
| TH27478 | 14028 *argW*::Zeo^R^-PmCherry *attB*::Km^R^-P*_fliF_*-YFP Δ*galK*::Ap^R^-P*_fliC_*-CFP Δ*prgH74* Δ*ssaN109* Δ*flgHI958* Δ*flk*-7755 Δ*fliK6620 motA8739* Δ*fliC7716* *fljB^enx^* *vh2* |  |
| TH27451 | 14028 *argW*::Zeo^R^-PmCherry *attB*::Km^R^-P*_fliF_*-YFP Δ*galK*::Ap^R^-P*_fliC_*-CFP Δ*prgH74* Δ*ssaN109* Δ*flgM5628*::FRT Δ*flgHI958* *motA8739* Δ*fliC7716* *fljB^enx^* *vh2* |  |

### Table S2. Oligonucleotides used in this study

| Name | Sequence (from 5’- to 3’-) |
| --- | --- |
| 1874-flhBCLtetR | gatggaagatgtgccgaaagcggacgtcattgtcactaacttaagacccactttcacatt |
| 1910-flhB375tetA | cgcgaccagattagccatcagtattcttctcgttcataaactaagcacttgtctcctg |
| 1911-flhBccDel-A | gcgcagcgccgcatgatggaagatgtgccgaaagcggacgtcattgtcactaactaattt |
| 1912-flhBccDel-B | cgcagcatcgcgaccagattagccatcagtattcttctcgttcataaattagttagtgac |
| 4543-fliKfullbla_rv | gatcttcagcatcttttactttcaccagcgtttctgggtgggcgaagatatccactgcgc |
| 7516-FliK278 | gtcatgttatttacgcgtcag |
| 8283-galK-sCFP3A | cgcggtcagcgacatccattttcgcgaatccggagtaTAAaaAGGTCTAGGCGGCGCCTA |
| 8284-galK-amp-cfp3A | cctgctccttgtgacggtttgcatacataaaaggtttcTTACCAATGCTTAATCAGTGAGGC |
| 8285-ybhCvenusNB | gcctgaaaaggaactttttaccttttcgccttcccgtttcgtGGCAGCAAAACCCGTACC |
| 8286-ybhC venusNBKan | agttaaatgacatccattgaagcctgcttttttatactaagttgaGCTTGGATTCTCACC |
| 8309-attB-Km_Prom-venusNB-tetR | acctataaaaataggcgtatcacgaggccctttcgtcttgac TTAAGACCCACTTTCACATT |
| 8315-galKtetR-scCFP3A | atttgaatgtatttagaaaaataaacaaataggggttccgcgTTAAGACCCACTTTCACATT |
| 8331-galK-tetA-CFP | ggtctagactccttactaaagttaaacaaaattattatcaatCTAAGCACTTGTCTCCTG |
| 8458-galK-PFliCsal | atttgaatgtatttagaaaaataaacaaataggggttccgcgGTTCTTTGTCAGGTCTGTC |
| 8459-attB-Km_PfliF-salm-venusfw | actcatatgtatatctccttcttaaagttaaacaaaattatt GGATTCGCGCGTAGGCGA |
| 8460-attB-Km_PfliFsal-venusrv | cctataaaaataggcgtatcacgaggccctttcgtcttgac CGTCGACTGCGAGTGC |
| 9083-new-attB-km-promvenusNBtetA | actcatatgtatatctccttcttaaagttaaacaaaattattCTAAGCACTTGTCTCCTG |
| 9142-galK-PFliC-RBSforCFP | ggtctagactccttactaaagttaaacaaaattattatcaatCGCAGACCGGAAGACAGA |
| 9305-attB-Cm-rv | tgagcttggattctcaccaataaaaaacgcccggcggcaaccCACTCATCGCAGTACTGTTGTAT |
| 9306-attB-Cm-fw | tttcttagacgtcggaattgccagctggggcgccctctggTCCTGGTGTCCCTGTTGATac |
| 9318-FliQtetR | tctcgccctggctgcgccgctgttactcgtcgcgctgattttaagacccactttcacatt |
| 9319-FliQtetA | aacgataattgcgatgaataccgcgacgattttagggataaactaagcacttgtctcctg |
| 9320-FliQ-AA31-50dopped | gttactcgtcgcgctgattaccggcctcattatcagcatcttgcaggccgcgactcagattaatgaaatgacgctgtcgtttatccctaaaatcgtcgcg |
| 9321-fliQ50-fill | cgcgacgattttagggat |
| 9322-FliQ-30fw | agtcgctctcgccctggctgcgccgctgttactcgtcgcgctgat |
| 9323-FliQ-50-rev | ggcaacgataattgcgatgaataccgcgacgattttagggat |

**Table S3. Number of cells expressing class 2 promoter and 3 promoter activity in individual bacterial cells, in different genetic backgrounds**

| Strain Number | Relevant Genotype  (all strains contain P*_fliF_*-*yfp* P*_fliC_*-*cfp*) | Number of cells expressing YFP | | | Number of cells expressing CFP | | | %  Class 3/2 |
| --- | --- | --- | --- | --- | --- | --- | --- | --- |
|  |  | Rep1* | Rep2 | Rep3 | Rep1 | Rep2 | Rep3 |  |
| TH28049 | wild type | 250 | 289 | 453 | 250 | 287 | 452 | 100 |
| TH27343 | *flgM* null | 289 | 295 | 269 | 289 | 295 | 270 | 100 |
| TH28052 | Δ*flk* | 340 | 293 | 241 | 388 | 289 | 239 | 99 |
| TH28050 | Δ*flgHI* | 609 | 134 | 273 | 1 | 0 | 0 | <0.01 |
| TH28051 | *flhB*_ΔCCD_ | 434 | 225 | 356 | 8 | 4 | 6 | 1.8 |
|  |  |  |  |  |  |  |  |  |
| TH28054 | Δ*flk* *flhB*_ΔCCD_ | 210 | 245 | 199 | 4 | 5 | 3 | 1.8 |
| TH29314 | Δ*flk* *flhB*_(N269A)_ | 457 | 478 | 370 | 7 | 7 | 6 | 1.5 |
|  |  |  |  |  |  |  |  |  |
| TH28053 | Δ*flgHI* Δ*flk* | 398 | 435 | 572 | 78 | 84 | 126 | 20.5 |
| TH28055 | Δ*flgHI flhB*_ΔCCD_ | 267 | 323 | 364 | 0 | 1 | 1 | 0.3 |
| TH29315 | Δ*flgHI flhB*_(N269A)_ | 467 | 553 | 540 | 0 | 0 | 0 | <0.01 |
|  |  |  |  |  |  |  |  |  |
| TH28056 | Δ*flgHI* Δ*flk* *flhB*_ΔCCD_ | 236 | 280 | 188 | 5 | 6 | 3 | 2 |
| TH29316 | Δ*flgHI* Δ*flk* *flhB*_(N269A)_ | 248 | 382 | 368 | 0 | 1 | 1 | <0.5 |
|  |  |  |  |  |  |  |  |  |
| TH29317 | *flhD*C** *flhB*_ΔCCD_ Δ*flk* | 341 | 252 | 353 | 275 | 198 | 285 | 90 |
| TH29318 | *flhD*C** *flhB*_ΔCCD_ Δ*flgHI* | 680 | 288 | 212 | 1 | 0 | 0 | 0.1 |
| TH29319 | *flhD*C** *flhB*_ΔCCD_ Δ*flk* Δ*flgHI* | 462 | 475 | 453 | 458 | 469 | 538 | 99 |
|  |  |  |  |  |  |  |  |  |
| TH29357 | *flhD*C** Δ*flhB* Δ*flk* | 366 | 289 | 295 | 0 | 0 | 0 | <0.01 |
| TH29358 | *flhD*C** Δ*flhB* Δ*flgHI* | 250 | 311 | 439 | 0 | 0 | 0 | <0.01 |
| TH29359 | *flhD*C** Δ*flhB* Δ*flk* Δ*flgHI* | 431 | 392 | 377 | 0 | 0 | 0 | <0.01 |
|  |  |  |  |  |  |  |  |  |
| TH27356 | Δ*flgHI* | 842 | 723 | 597 | 3 | 2 | 1 | <0.5 |
| TH27359 | Δ*flgHI* Δ*flk* | 179 | 214 | 264 | 37 | 40 | 51 | 20 |
| TH27477 | Δ*flgHI flhB*_ΔCCD_ | 232 | 345 | 216 | 0 | 0 | 0 | <0.5 |
| TH27476 | Δ*flgHI* Δ*flk flhB*_ΔCCD_ | 216 | 277 | 224 | 2 | 2 | 2 | 0.8 |
| TH27478 | Δ*flgHI* Δ*flk* Δ*fliK* | 296 | 256 | 292 | 0 | 0 | 0 | <0.01 |
| TH27451 | Δ*flgHI* Δ*flgM* | 127 | 144 | 163 | 149 | 166 | 198 | <100 |

* Replicates (Rep1, 2, 3) were conducted using three independent biological cultures. Complete genotypes can be found in Table S1.

**Table S4. Strains used in Figure 7 and supplemental Figure 5:**

**FlgM-Bla strains:**

All strains contain *flgM6407*::*bla* Δ*flgB-L8735* *fliA5225* Δ*fliB-T7771 fljB^enx^ vh2*

|  | WT | FliP (I95N) | FliQ (G32D) | FliR (Q210 stop) | FliR (V215D) | FliR (T221DUP) |
| --- | --- | --- | --- | --- | --- | --- |
| *flk*^+^ | TH29405 | TH29427 | TH29431 | TH29439 | TH29443 | TH29447 |
| Δ*flk* | TH29406 | TH29428 | TH29432 | TH29440 | TH29444 | TH29448 |
| Δ*fliK* | TH29684 | TH29451 | TH29455 | TH29463 | TH29467 | TH29471 |
| Δ*fliK* Δ*flk* | TH29685 | TH29452 | TH29456 | TH29464 | TH29468 | TH29472 |

**FlgE-Bla strains:**

All strains contain *flgE6569*::*bla* Δ*flgBC6557 fliA5225* Δ*fliB-T7771 fljB^enx^ vh2*

|  | WT | FliP  (I95N) | FliQ (G32D) | FliR  (Q210 stop) | FliR (V215D) | FliR (T221DUP) |
| --- | --- | --- | --- | --- | --- | --- |
| *flk*^+^ | TH29407 | TH29429 | TH29433 | TH29441 | TH29445 | TH29449 |
| Δ*flk* | TH29408 | TH29430 | TH29434 | TH29442 | TH29446 | TH29450 |
| Δ*fliK* | TH29686 | TH29453 | TH29457 | TH29465 | TH29469 | TH29473 |
| Δ*fliK* Δ*flk* | TH29687 | TH29454 | TH29458 | TH29466 | TH29470 | TH29474 |

**FliK-Bla strains:**

All strains contain *fliK7582*::*bla* *flhB7152*(N269A) Δ*flgB-L8735* *fliA5225* Δ*fliB-T7771 flhD8070 flhC8092 fljB^enx^ vh2*

|  | WT | FliP I95N | FliQ (G32D) | FliR Q210 stop | FliR T221DUP | FliR V215D | FliK P296L | FliK Δ311-320 |
| --- | --- | --- | --- | --- | --- | --- | --- | --- |
| *flk*^+^ | TH30353 | TH30354 | TH30355 | TH30357 | TH30359 | TH30358 | TH30405 | TH30403 |
| Δ*flk* | TH30360 | TH30361 | TH30362 | TH30364 | TH30366 | TH30365 | TH30406 | TH30404 |

**Supplemental Figure Legends**

**Supplemental Figure 1.**The deletion of *flk* has no apparent effect on *Salmonella* motility.

Twenty independent assays comparing the Δ*flk* strain (TH14607) to the parental *flk*⁺ LT2 strain showed no statistically significant difference in motility (Welch’s t-test), indicating that loss of *flk* does not measurably impact motility.

**Supplemental Figure 2.** A bacterial two hybrid screen for interaction between either Fluke or FliK with the cytoplasmic domain of FlhA (FlhA_C_) indicates a positive interaction phenotype (Lac^+^ pink color formation). Lane 1= pUT18C-FliK x pKNT25 empty vector; lane 2= pUT18C empty vector x pKNT25-FlhA C-terminus; lanes 3 and 4 = pUT18C-Fluke x pKNT25-FlhA C-terminus; lane 5= pUT18C-Fluke x pKNT25 empty vector; lane 6= pUT18C empty vector x pKNT25-FlhA C-terminus; lanes 7 and 8 = pUT18C-FliK x pKNT25-FlhA C-terminus.

**Supplemental Figure 3.** The cleavable C-terminal domain of FlhB is not detected in the cell supernatant, just after the secretion specificity switch has occurred. Cells (1L) were grown to OD 0.5. Cells were pelleted, washed once and resuspended into 20 ml of media containing arabinose and induced for 15 mins. Supernatant was concentrated to 200 μl and 20 μl/lane was loaded.

**Supplemental Figure 4.** Motile revertants of Δ*fliK* Δ*flk* double mutant strain in the *flhD***C** background. **A.** Motility relative to the parent TH29848 (labeled red) and a *fliK*^+^ *flk*^+^ motile strain showing that motile revertants do not exhibit full “wild-type” motility after 4 hours incubation in soft agar motility plates at 37°C. **B.** Motility phenotypes relative to the parent strain (red), TH29848, after 8 hours incubation in soft agar medium at 37°C. **C.** Double-mutants with increased motility shown relative to the original motile revertant phenotype (red) after 10 hours incubation in soft agar motility plates at 37°C. **D.** These are motility revertants with only slight increased motility phenotypes after 16 hours incubation on soft agar motility plates at 37°C.

**Supplemental Figure 5.** Levels of early (FlgE-Bla) and late (FlgM-Bla) substrate secretion in the FlhB-bypass mutants in the presence and absence of Fluke, and/or FliK. Parent strain numbers (*flk*^+^) are indicated in the figure. Genotypes of all strains used are listed in Table S4.
